# Phase I trial of vorinostat and doxorubicin in solid tumours: histone deacetylase 2 expression as a predictive marker

**DOI:** 10.1038/sj.bjc.6605293

**Published:** 2009-09-08

**Authors:** P N Munster, D Marchion, S Thomas, M Egorin, S Minton, G Springett, J-H Lee, G Simon, A Chiappori, D Sullivan, A Daud

**Affiliations:** 1 Division of Hematology and Oncology, University of California, San Francisco, CA 94143, USA; 2 H. Lee Moffitt Cancer Center and Research Institute, Tampa, FL 33647, USA; 3 Department of Molecular Therapeutics/Drug Discovery Program, University of Pittsburgh Medical Center, Pittsburgh, PA 15213, USA

**Keywords:** DNA topoisomerases, type II, histone deacetylases, doxorubicin, clinical trials, phase I, chromatin decondensation

## Abstract

**Background:**

Histone deacetylase inhibitors (HDACi) can sensitise cancer cells to topoisomerase inhibitors by increasing their access and binding to DNA.

**Methods:**

This phase I trial was designed to determine the toxicity profile, tolerability, and recommended phase II dose of escalating doses of the HDACi vorinostat, with weekly doxorubicin.

**Results:**

In total, 32 patients were treated; vorinostat was dosed at 400, 600, 800, or 1000 mg day^−1^ on days 1–3, followed by doxorubicin (20 mg m^−2^) on day 3 for 3 of 4 weeks. Maximal tolerated dose was determined to be 800 mg day^−1^ of vorinostat. Dose-limiting toxicities were grade 3 nausea/vomiting (two out of six) and fatigue (one out of six) at 1000 mg day^−1^. Non-dose-limiting grade 3/4 toxicities included haematological toxicity and venous thromboembolism. Antitumor activity in 24 evaluable patients included two partial responses (breast and prostate cancer). Two patients with melanoma had stable disease for ⩾8 months. Histone hyperacetylation changes in peripheral blood mononuclear and tumour cells were comparable. Histone hyperacetylation seemed to correlate with pre-treatment HDAC2 expression.

**Conclusion:**

These findings suggest that vorinostat can be combined with weekly doxorubicin in this schedule at a dose of 800 mg day^−1^. The HDAC2 expression may be a marker predictive of HDAC inhibition. Antitumor activity of this regimen in breast cancer, prostate cancer, and melanoma seems interesting.

Histone deacetylase (HDAC) inhibitors, agents that modulate chromatin plasticity, are undergoing extensive clinical and non-clinical evaluation. Acetylation and deacetylation of histones or non-histone protein targets are directly or indirectly associated with tumour evolution and tumour progression (Grunstein, 1997; Marks *et al*, 2001). The HDAC inhibitors have been studied in a wide range of cancers either alone or in combination with other anticancer agents. The first HDAC inhibitor to be approved was vorinostat (Zolinza, Merck, Whitehouse Station, NJ, USA), a hydroxamic acid-type HDAC inhibitor, for cutaneous T-cell lymphoma (Kelly *et al*, 2003, 2005; O’Connor *et al*, 2006; Duvic *et al*, 2007).

The HDAC inhibitors can potentiate the cytotoxicity of anthracycline-type topoisomerase (topo) II inhibitors such as doxorubicin, epirubicin, and mitoxanthrone (Tsai *et al*, 2000; Johnson *et al*, 2001; Kurz *et al*, 2001; Marchion *et al*, 2005a, 2005b, 2005c; Munster *et al*, 2007). Topo II inhibitors induce DNA strand breaks by binding to DNA, stabilizing the topo II-DNA complex, and inhibiting the re-ligation of DNA strands during replication. HDAC inhibitor-induced chromatin decondensation increases the binding of topo II inhibitors to their DNA substrate (Marchion *et al*, 2005a, 2005b, 2005c). Xenograft studies have suggested that synergy between HDAC and topo II inhibitors is dose-dependent and requires exposure to the HDAC inhibitor for at least 48 h before the topo II inhibitor (Johnson *et al*, 2001; Kurz *et al*, 2001; Marchion *et al*, 2004). On the basis of these and other preclinical data, a phase I trial with the HDAC inhibitor valproic acid (VPA) and epirubicin was performed with promising results (Munster *et al*, 2007, 2009). There are clear limitations, however, to VPA: it has low potency and significant neurological toxicity. We initiated this study, therefore, to determine whether the potency and low toxicity of the hydroxamic acid derivative vorinostat could be exploited in combination with weekly doxorubicin. As the subtype specificity of the HDAC target of vorinostat is not known, we undertook extensive sampling of tumour cells and peripheral blood mononuclear cells (PBMCs) to determine whether the response could be predicted based on expression of specific HDAC subtype.

## Materials and methods

### Trial design

This trial was approved by the Protocol Review Committee, the Institutional Review Board at the Moffitt Cancer Center, and the Cancer Therapy Evaluation Program (the study sponsor) (Clinicaltrials.gov identifier NCT00331955). Vorinostat was administered orally on days 1, 2, and 3 with a planned vorinostat dose escalation of 400, 600, 800, and 1000 mg given in two divided daily doses. Fixed doses of doxorubicin 20 mg m^−2^ (Adriamycin, Pfizer, New York, NY, USA) were infused on day 3, 4 h after the last vorinostat dose. Treatment was given weekly 3 out of 4 weeks; a cycle consisted of 4 weeks. A maximum of six cycles (24 weeks) of doxorubicin was allowed. In patients with demonstrated benefit, doxorubicin was then stopped and patients were treated with vorinostat only. The dose escalation followed a conventional dose escalation scheme. In the absence of dose-limiting toxicities (DLTs), cohorts were limited to three evaluable patients.

### Patients

Eligible patients (age ⩾18 years) had to have histologically confirmed advanced solid tumour malignancies with an ECOG performance status of 0–2 and adequate organ function (haemoglobin >9.0 g per 100 ml, white blood cell >3000 cells per mm^3^, ANC >1500 cells per mm^3^, platelets >100 000 cells per mm^3^, creatinine ⩽2 mg per 100 ml, bilirubin ⩽1.5 mg per 100 ml, and liver enzymes within 1.5 × the upper level of institutional normal). Patients with earlier anthracycline exposure, an ejection fraction of <50% by multigated acquisition scan (MUGA) or echocardiogram, a history of long QT syndrome, or corrected QTc (QTc) prolongations >470 ms at baseline were excluded.

### Treatment and toxicity assessment

Baseline safety and toxicity evaluations included history and physical examination, 12-lead ECG, and complete blood count with differential, metabolic, hepatic, and renal function assessments. These tests were repeated weekly during the first cycle and then every 4 weeks in asymptomatic patients. Cardiac function was assessed by MUGA or echocardiogram every two cycles. A 12-lead ECG was performed before vorinostat treatment and on day 3 in cycle 1. Day 3 ECGs in subsequent cycles were only performed if the day 1 ECG showed any abnormalities or prolongations of the QT interval. Tumour assessment by computer tomography, bone scan, or MRI scan was performed at baseline and repeated after every two cycles (8 weeks). Toxicities were captured in all patients and graded by the Common Terminology Criteria for Adverse Events (CTCAE v3). The DLTs were defined in cycle 1 as any grade ⩾3 non-haematological toxicities; grade 3 thrombocytopenia lasting for >7 days or associated with bleeding; or any grade 4 haematological toxicity, with the exception of grade 4 neutropenia or leucopenia for <8 days. Furthermore, any grade 2 toxicity that prevented dose delivery in cycle 1 was considered dose limiting. Patients in the dose escalation cohorts who did not complete the first cycle and were not evaluable for toxicity had to be replaced. Toxicities were captured in all patients who received at least one dose of vorinostat; however, patients receiving less than one full cycle of treatment and who were not evaluable for DLTs were replaced.

Patients were considered evaluable for response if they had at least two cycles of treatment or were taken off study early for progression. Tumour response was graded by the response evaluation criteria in solid tumours (RECIST) (Therasse *et al*, 2000). The correlation between variables was tested for statistical significance using Spearman's correlation coefficients with two-sided *P*-values at a 0.05 significance level (SAS version 9.1, SAS Institute, Inc., Cary, NC, USA). Spearman's correlation coefficient methods were used to estimate correlations between two variables and to perform the test of significance of the estimated correlation.

### Pharmacokinetic studies

Limited assessment of vorinostat plasma levels was obtained with high-performance liquid chromatography (HPLC) at time of tumour biopsies immediately before doxorubicin infusion (time 0) and 2 and 48 h after doxorubicin infusion. A total of 5 ml whole blood from a peripheral vein were collected in a Red Top Vacutainer tube and refrigerated at 4°C. The clotted samples were centrifuged at 2000 **g** for 15 min at 4°C and stored at −70°C. All pharmacokinetic samples were analysed by Dr M Egorin (University of Pittsburgh) by HPLC methods as previously described (Parise *et al*, 2006).

### Correlative studies

The PBMCs were isolated with Ficoll centrifugation (Ficoll-Paque, GE HealthCare, Uppsala, Sweden) and adhered to glass slides using cytospin funnels or collected in freezing media in liquid nitrogen for western blot analysis. Tumour tissue was obtained by fine needle aspirations of skin and subcutaneous lesions or core biopsies of deeper structures under image guidance. The PBMC and tumour samples were rapidly fixed with 5–95% acetic acid in ethyl alcohol for 1 min. Pre- and post-treatment slides were stained with either anti-acetylated histone H3 or H4 (Upstate Biotechnology, Lake Placid, NY, USA; polyclonal, 1 : 200), HDAC2 (Upstate Biotechnology; monoclonal), HDAC6 (Abcam, Cambridge, MA, USA; polyclonal, 1 : 200), topo II*α*, topo II*β*, and heterochromatin protein 1 (HP-1) (all Upstate Biotechnology; polyclonal 1 : 200). Slides were simultaneously counterstained with anti-pan histone 3 (Upstate Biotechnology; polyclonal, or BD Biosciences, San Jose, CA, USA; monoclonal, 1 : 200) for 1 h. Respective proteins were then developed with anti-rabbit Alexa-Fluor 546 and anti-mouse Alexa-Fluor 488 (Molecular Probes, Eugene, OR, USA) and bisbenzimide (0.5 mg ml^−1^) for 1 h. All pre- and post-treatment PBMC and tumour samples were evaluated by immunofluorescence confocal microscopy. Resultant images were analysed as previously described (Marchion *et al*, 2005a, 2005c; Munster *et al*, 2009). All PBMC samples were processed for western blot analysis, and membranes were probed with the respective antibodies as outlined for immunofluorescence staining: anti-acetylated histone H4 or H3, topo II*α*, topo II*β*, HP-1, HDAC2, and HDAC6 (all rabbit polyclonal, Upstate Biotechnology). Pre- and post-vorinostat pan H3 expression served as internal control.

## Results

### Treatments and toxicity

In total, 32 patients received at least one dose of study treatment. A total of 30 patients completed at least one cycle of treatment, but two patients in the first cohort could not be evaluated for toxicity and were replaced: one patient required blood transfusion for bleeding of a pre-existing wound and a second patient was found to have brain metastases on day 8. Two patients at the maximally administered dose of 1000 mg vorinostat and one patient at the dose expansion cohort (800 mg) withdrew consent due to side-effects after one cycle. Patient demographics and tumour characteristics for all 32 patients are shown in Table 1. Overall, 24 patients completed at least two cycles of treatment and were evaluable for response.

### DLTs and serious adverse events

The first DLT was observed in the 800-mg cohort (Table 2). A breast cancer patient with extensive bone marrow involvement and multiple earlier cytotoxic therapies experienced grade 3 thrombocytopenia, which required protocol prespecified dose modification. No further platelet-related DLTs were observed during the first cycle of the study in any other patient, but two more patients had grade 3 thrombocytopenia at the 800 and 1000 mg vorinostat doses in subsequent cycles. At the maximally administered dose of 1000 mg day^−1^, two patients experienced DLTs: one patient had grade 3 fatigue (one out of six) and one patient had grade 2 nausea/vomiting in week 1 and week 2 despite optimal supportive care that prevented continued treatment (one out of six). Both DLTs resolved after treatment interruption, but both patients withdrew consent (Table 2). At the dose expansion cohort, one patient (1 out of 12) experienced a rise in the liver enzymes, resulting in grade 3 toxicity. Vorinostat was reduced to 600 mg daily for 3 days, and the patient did not experience further changes in liver function during the first cycle. The patient withdrew consent after 4 weeks for personal reasons. All together, 18 patients were treated with 800 mg daily for 3 days and 2 patients experienced DLTs. All four of the DLTs observed in the study occurred in female patients.

### Haematological toxicities

Non-dose-limiting grades 3 and 4 neutropenia was seen in 8 out of 32 patients (25%) (Table 3). Grade 3 thrombocytopenia occurred in 3 out of 32 (9%) patients, including the patient with grade 3 thrombocytopenia meeting the DLT criteria. Treatment-related anaemia was observed in one (3%) patient.

### Non-haematological toxicities

As described above, DLTs included 2 out of 18 patients on the 800 mg daily dose and 2 out of 6 at the 1000 mg total daily dose of vorinostat, given on days 1–3 of every week for 3 or 4 weeks followed by doxorubicin. In addition to the observed DLTs, a major toxicity was thromboembolic events occurring in 4 out of 32 patients (13%). These presented mainly as asymptomatic unilateral or bilateral pulmonary emboli diagnosed on restaging images. Although the temporary association with the study renders the pulmonary emboli likely study drug related, a clear attribution to either vorinostat, doxorubicin, or the combination cannot be attained in the absence of a control arm without vorinostat. A more precise attribution must be sought in future randomized studies. Other grade 3 and 4 toxicities observed in study but after the DLT period are depicted by cohort in Table 3. Predominant grade 2 toxicities included fatigue in 17 out of 32 (53%), anorexia in 15 out of 32 (47%), nausea/vomiting in 6 out of 32 (19%), diarrhoea in 5 out of 32 (16%), alopecia in 8 out of 32 (25%), hyperglycaemia in 3 out of 32 (9%), and somnolence in 1 out of 32 (3%) patients.

### Cardiac toxicities

Although this protocol was not designed to determine cardiac toxicities of vorinostat, limited cardiac evaluations were performed for safety. All patients were followed with MUGA scans or echocardiograms obtained every 8 weeks while on study. The median ejection fraction at baseline was 61.5% (95% confidence interval 60.1–62.8%). A median change of +2% (95% confidence interval 0.7–3.3%) was observed over the course of the study for all patients, and no grade 2 or higher changes in left ventricular ejection fractions were observed in individual patients. On the basis of reports of prolongations in QTc intervals seen in studies with this and other HDAC inhibitors, patients were screened for long QTc intervals and all patients had an ECG on days 1 and 3 in cycle 1. Further ECGs were obtained only on day 1 of each cycle, but not on day 3 unless the QTc interval on day 1 was >450 ms. One patient treated in the 1000 mg vorinostat cohort and two patients in the 800 mg cohort experienced a grade 2 QTc prolongation on day 3. A grade 2 QTc (475 ms) was observed in a fourth patient on day 1 of cycle 2, which persisted through day 3 (481 ms). No intervention other than electrolyte replenishment was needed in these patients.

### Response

Patients experiencing clinical or radiological tumour progression before completion of two cycles were included in the evaluation and considered ‘progression’ (Table 2). Overall, 8 of 32 patients were not evaluable, several patients withdrew consent after one cycle, and one patient presented with brain metastases on day 8 of cycle 1 and received only 1 week of treatment. Of 24 evaluable patients, confirmed partial responses by radiological RECIST or by prostate-specific antigen assessment were seen in 2 out of 24 (8%) patients lasting for 8 months. Two patients with melanoma met the criteria for stable disease and minor response for 8 months. After receiving the prespecified maximum of six cycles of doxorubicin, all of the four patients with either a response or clinical benefit for more than 6 months progressed on vorinostat alone after doxorubicin was stopped, suggesting a benefit from the combination rather than vorinostat. The disease types in which a benefit was observed, including breast (one), melanoma (two), prostate (one), were similar to those seen in the trial evaluating VPA and epirubicin (Munster *et al*, 2007).

### Pharmacodynamic and correlative studies

**Figure 1:**
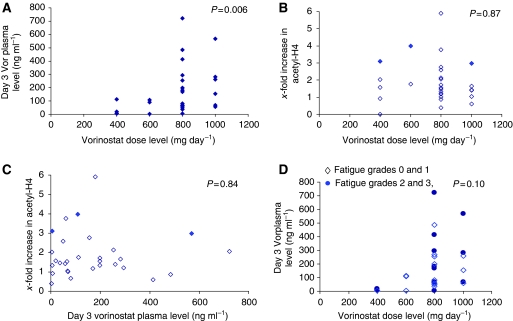
Scatter diagrams of day 3 vorinostat dose, plasma levels, and histone acetylation. (**A**) Vorinostat (Vor) plasma levels in response to various doses (mg day^−1^). (**B**) Change in H4 histone acetylation by western blot at different vorinostat doses. (**C**) Change in H4 histone acetylation at different vorinostat plasma levels. Closed diamonds (♦) depict patients with objective response or clinical benefit (>6 months). (**D**) Vorinostat plasma levels at different doses for patients with grade 0 and 1 (⋄) or grade 2 and 3 fatigue (•).

Preclinical studies suggest that a 48-h exposure to an HDAC inhibitor will induce chromatin decondensation and modulation of topo targets. The pharmacokinetic studies were limited and directed towards defining the vorinostat dose levels at time of doxorubicin exposure and correlating them with the effects on deacetylase inhibitor targets. Vorinostat plasma levels were obtained on day 3 at the time of the tumour biopsy and after a 3- to 4-h time interval after the last vorinostat dose. Vorinostat plasma levels showed a significant increase (*n*=32, *P*=0.006) with dose, albeit with wide interpatient variability (Figure 1A). The median vorinostat dose of the 18 patients treated with the maximal tolerated dose of 800 mg day^−1^ was 174.6±30.7 ng ml^−1^ and equalled 0.66±0.12 *μ*M, similar to concentrations (0.5–1 *μ*M) required for *in vitro* synergy (Marchion *et al*, 2004).

Although vorinostat dose and levels were correlated, neither histone H4 nor H3 acetylation in PBMCs was correlated with vorinostat dose or vorinostat plasma level (Figure 1B and C). Of the four patients with a response or a clinical benefit of >6 months, three patients showed high histone acetylation. The fourth patient did not have sufficient PBMCs collected for analysis. The patient with a 5.9-fold increase in acetyl-H4 was not evaluable for response. As reported above, grade 2 or greater fatigue was seen in >50% of the patients. Fatigue was not correlated with dose or vorinostat plasma levels obtained in cycle one (Figure 1D and data not shown).

**Figure 2:**
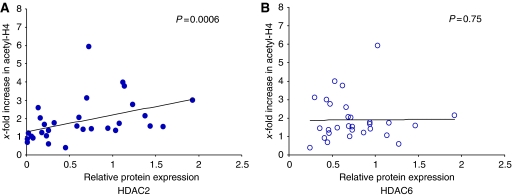
Correlation of Histone deacetylase (HDAC) 2 and HDAC6 expression with histone acetylation. Scatter plot of relative baseline protein expression of HDAC2 (**A**, closed circles) and HDAC6 (**B**, open circles) plotted against change (*x*-fold increase) in histone acetylation. HDAC2 (*P*=0.0006) but not HDAC6 (*P*=0.75) was positively correlated with a change in histone H4 acetylation.

Vorinostat affects several HDAC enzyme targets, and preclinical and clinical data with VPA have suggested that HDAC2 may be one of the most relevant targets and its expression may be associated with the degree of H4 acetylation (Marchion *et al*, 2009). Vorinostat, a hydroxamic acid-type HDAC inhibitor, also affects HDAC6, which is a distinguishing factor between selective and non-selective HDAC inhibitors. We found that baseline expression of HDAC2 but not HDAC6 was associated with increased acetylation of histone H4 (Figure 2A and B). Several investigators have reported a decrease in HDAC2 expression during exposure to HDAC inhibitors; in this study, we did not find any changes in pre- and post-treatment HDAC2 expression (median change in HDAC2: +4% (95% confidence interval: −8 to +20% in PBMCs)).

Preclinical studies have suggested that exposure of cells to an HDAC inhibitor is associated with histone hyperacetylation and subsequent chromatin decondensation. The change in chromatin plasticity is associated with a depletion of chromatin remodelling proteins such as HP-1, structural maintenance of chromatin proteins (SMC1-5), and DNA methyltransferase 1 (Marchion *et al*, 2005b). Furthermore, depletion of topo II*α* has been associated with chromatin decondensation (Adachi *et al*, 1991; Grue *et al*, 1998; Durrieu *et al*, 2000; Cuvier and Hirano, 2003; Marchion *et al*, 2005b).

**Figure 3:**
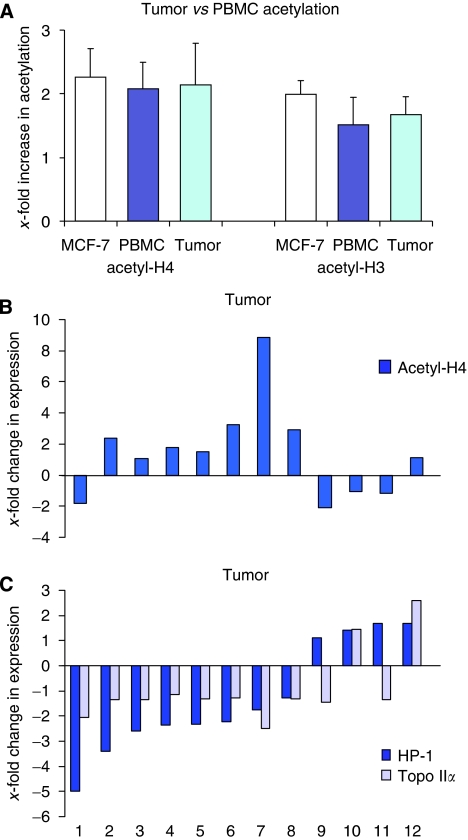
Changes in histone acetylation, heterochromatin protein 1 (HP-1), and topoisomerase (topo) II*α* expression in tumours and peripheral blood mononuclear cells (PBMCs). (**A**) Bar graph of average change in histone H4 and H3 acetylation in cultured MCF-7 cells (1 *μ*M vorinostat), patient-derived PBMCs, and tumour cells (s.e. bars: confidence intervals) by immunofluorescence. (**B**) Increase in acetylated histone H4 and (**C**) decrease in HP-1 and topo II*α* expression in individual patients (1–12) treated at the dose expansion level by immunofluorescence.

Pre- and post-vorinostat PBMC samples were obtained in all patients, but tumour biopsies were obtained in the 12 patients treated at the dose expansion cohort. The average increase in H3 and H4 acetylation induced by vorinostat was comparable in tumours and PBMCs (Figure 3A). The effects were further comparable with histone acetylation seen in cultured MCF-7 cells treated with 1 *μ*M vorinostat for 48 h. Induction of tumour H4 acetylation was observed in 8 out of 12 patients (Figure 3B). Vorinostat effects on downstream targets associated with chromatin decondensation, depicted as depletion of HP-1 and topo II*α* expression, were observed in 8 out of 12 (66%) and 10 out of 12 patients (83%) respectively (Figure 3C).

## Discussion

Although HDAC inhibitors have shown efficacy in haematological malignancies (Marshall *et al*, 2002; Sandor *et al*, 2002; Byrd *et al*, 2005; Kelly *et al*, 2005; Garcia-Manero *et al*, 2008), their single-agent activity in solid tumours has been limited (Reid *et al*, 2004; Luu *et al*, 2006; Stadler *et al*, 2006; Blumenschein *et al*, 2008). This study describes a sequenced combination with the HDAC inhibitor vorinostat given for 48 h followed by an anthracycline-type topo II inhibitor, doxorubicin (Table 1). This sequence was based on cell culture, *in vivo* models, and a previous phase I clinical trial evaluating the HDAC inhibitor VPA in combination with epirubicin (Munster *et al*, 2007). Preclinical models had shown that HDAC inhibitor-induced histone hyperacetylation and chromatin decondensation correlated with increased binding of topo II inhibitors to the DNA substrate. *In vitro* data also suggested that histone acetylation was necessary but not sufficient for chromatin decondensation and required HDAC inhibitor-induced depletion of chromatin remodelling proteins. HDAC inhibitor treatment was further associated with topo II*α* depletion and recruitment of topo II*β* (Marchion *et al*, 2004, 2005a, 2005b, 2005c).

The HDAC inhibitor used in this study is a hydroxamic-type HDAC inhibitor with broader effects on HDAC enzymes, including Class IIB and higher potency than VPA. The approved dose of vorinostat (as a single agent) is 400 mg day^−1^, which is used in most studies (Kelly *et al*, 2005). Higher doses have been explored, but the cumulative toxicities, which include thrombocytopenia, nausea, vomiting, anorexia, and fatigue, limit higher doses when given daily for prolonged periods. In this study, we used vorinostat to increase the efficacy of topo II inhibitors. Hence, the tolerability of higher drug exposure, but at a shorter interval, was explored. A weekly doxorubicin dosing was employed with the intent to maximise exposure to vorinostat. We show that, when given for 3 days before a weekly administration of doxorubicin (20 mg m^−2^) for 3 of 4 weeks, the maximum tolerated dose, as well as recommended phase II dose for the combination, was 800 mg (400 mg twice daily for five doses) in patients with advanced solid tumour malignancies and a median of 2 before treatment regimens for metastatic disease (Tables 1,2,3), which is higher than the single-agent recommended dose. Fatigue (grade 2 and higher) was seen in more than 50% of the patients beyond cycle 1 and was also seen when doxorubicin was stopped. All reported toxicities resolved rapidly after discontinuation of vorinostat. The observed fatigue was not correlated with vorinostat doses or plasma level, and no association was seen between histone acetylation in PBMC and fatigue (Figure 1D and data not shown). Thromboembolic events, pulmonary emboli in all four patients, occurred more frequently than expected. Although thromboembolic events have been associated with anthracyclines and cancer, the risk associated with vorinostat is still under investigation and will require randomized trials to determine the true incidence. Anticoagulation should be considered in patients at risk.

In this trial, 24 patients were evaluable for efficacy. Of these, two patients had a partial response and two patients had stable disease beyond 6 months. All four of these patients progressed once the doxorubicin was stopped, suggesting the benefit was more likely associated with the combination rather than with vorinostat. The benefits seen in the patients with breast and prostate cancer may be attributed to the anthracycline alone; however, melanoma is not an anthracycline-sensitive disease (Lopez *et al*, 1984; Rosenthal *et al*, 1998). Only testing in randomized trials will be able to determine how much benefit the HDAC inhibitors contribute. The number of patients is small on this trial; however, those patients with a response or benefit have higher levels of histone acetylation irrespective of vorinostat plasma levels or dose (Figure 1B and C). The patient population with benefit from this therapy was similar to those seen with the initial proof of principal study involving VPA and epirubicin (Munster *et al*, 2007, 2009). Several patients (7 out of 32) withdrew consent after one cycle in this study, which is not uncommon in patients treated with multiple regimens; however, it may also suggest that the toxicities, although not dose limiting or grade 3 by CTCAE criteria, may be unacceptable to patients. In particular, fatigue has been commonly described in vorinostat trials. Fatigue and nausea were more common with vorinostat, whereas somnolence and neurovestibular symptoms were more commonly associated with VPA. Although the VPA-induced somnolence was troubling to many patients, the every 3-week administration may have rendered these short-lived toxicities more acceptable and did not lead to withdrawal of patients from the study. Emerging data suggest that differential inhibition of select HDAC enzymes is required for efficacy among different tissues, and much emphasis has been placed on developing selective HDAC inhibitors to improve efficacy. However, given the varying toxicities between VPA and vorinostat, the development of select HDAC inhibitors may also allow minimisation or narrowing of toxicities seen with the non-selective drugs.

One of the issues in monitoring the efficacy of HDAC inhibitors has been determining the most appropriate surrogate marker for monitoring the molecular effects of these agents. Furthermore, there are limited data on downstream or other on-target effects of vorinostat in solid tumour malignancies. Experimental data from cell culture and xenograft data have suggested that histone acetylation may occur as early as 30 min after exposure, and the depletion of chromatin remodelling genes and chromatin decondensation required a prolonged exposure (at least 24–48 h) to an HDAC inhibitor. Furthermore, our preclinical study showed synergistic interactions between HDAC and topo inhibitors in the setting of HDAC inhibitor-induced depletion of topo II*α* (Marchion *et al*, 2005c). In addition to the vorinostat-induced effects on H3 and H4 acetylation in PBMCs and tumour cells, we examined effects on a representative of chromatin remodelling proteins (HP-1) and topo II*α*. We found that HP-1 levels, which were assessed by immunofluorescence, were decreased in 8 out of 12 and topo II*α* levels in 10 out of 12 patients treated at the maximum tolerated dose, whereas histone H4 acetylation was increased in 8 out of 12 patients, mirroring preclinical *in vitro* and *in vivo* data.

There has been much emphasis on the distinction of selective *vs* non-selective drugs and whether selectiveness will infer efficacy. We have shown that, for chromatin remodelling, inhibition of HDAC2 is crucial (Marchion *et al*, 2009). In addition to these findings *in vitro*, we show in this study that pre-treatment expression of HDAC2 can predict histone hyperactelyation. These findings suggest that HDAC2 may be useful both as a response prediction biomarker and as a target for the development of isotype-specific inhibitors that could potentially achieve even higher-level inhibition without the toxicity seen in current generation compounds.

## Figures and Tables

**Table 1:** Patient characteristics (*n*=32)

*Gender*	
Female	17 (53%)
Male	15 (47%)
	
*Age, years*	
Median (range)	53 (24–83)
⩾65 years (%)	7 (22)
	
*Mean height, cm (range)*	170 (156–187)
Female	164 (156–172)
Male	178 (170–187)
	
*Mean weight, kg*	79 (48–127)
Female (range; BMI)	75 (48–127; 28.1)
Male (range; BMI)	84 (68–100; 26.5)
	
*Race/ethnicity*	
Caucasian	28 (88%)
African American	2 (6%)
Hispanic	2 (6%)
	
*Tumour histology*	
Melanoma	6 (19%)
Breast	5 (16%)
Lung cancer	6 (19%)
Sarcoma	3 (9%)
Colon	2 (6%)
Prostate	2 (6%)
Oesophageal cancer	3 (9%)
Other (pancreatic, endometrial, neuroendocrine, renal cell, bladder carcinoma)	5 (16%)
	
*ECOG performance status*	
0	26 (81%)
1	6 (19%)
	
*No. of earlier systemic metastatic regimens, median (range)*	2 (0–6)
No. of cytotoxic agents, median (range)	1 (0–4)
No. of targeted, hormonal or immunotherapy, median (range)	1 (0–6)
No. of patients with earlier radiation therapy (%)	11 (34%)

Abbreviation: BMI=body mass index.

**Table 2:** Treatment and responses

**Total daily vorinostat dose (mg day^−1^)**	**Single dose, every 12 h × 6**	**Doxorubicin, mg m^−2^ (day 3)**	**No. of patients (*N*=32)**	**No. of dose-limiting toxicities (evaluable: *N*=30)**	**Best response (evaluable: *N*=24) (partial response and stable disease ⩾12 weeks)**
*Dose escalation*					
400	200	20	5 (3 evaluable)		PR: 8 months (breast)
600	300	20	3		PR: 8 months (prostate) SD: 8 months (melanoma)
800	400	20	3+3	Thrombocytopenia (1/6)	SD: ⩾4 months (breast)
1000	500	20	3+3	Fatigue (1/6) Nausea/vomiting (1/6)	SD: 8 months (melanoma) SD (prostate)^a^
					
*Dose expansion*					
800	400	20	12	Elevated liver enzymes (1/12)	SD: ⩾4 months (oesophageal)

Abbreviations: PR=partial response; SD=stable disease.

a Patient withdrew consent after two cycles but remained stable.

**Table 3:** Grades 3 and 4 toxicities (all cycles)

**Toxicity**	**Dose escalation**				**Dose expansion**
Dose (*n*)	400 (3)	600 (3)	800 (6)	1000 (6)	800 (12)
Neutropenia	1	1	1	2	3
Thrombocytopenia	0	0	1	1	1
Anemia	0	1	0	0	0
Lymphopenia	0	0	0	0	2
Fatigue	0	0	0	1	0
Elevated liver enzymes	0	0	0	0	1
Dehydration	1	0	0	0	0
Nausea/vomiting	1	0	0	1	0
Syncope	0	0	0	1	0
Pulmonary emboli	1	0	1	1	1
